# Continuous glucose monitoring in paediatric residents with normal glucose metabolism: correlations to 24-hour duty schedule

**DOI:** 10.1007/s40200-026-01871-1

**Published:** 2026-03-03

**Authors:** Georgia Sotiriou, Anastasia Proimou, Paraskevi Panagopoulou, Zoi Tsimtsiou, Athanasios Christoforidis

**Affiliations:** 1 1st Department of Pediatrics, School of Medicine, Faculty of Health Sciences, Aristotle University, Ippokratio General Hospital, 49, Konstantinoupoleos str, Thessaloniki, 54642 Greece; 2 School of Medicine, Faculty of Health Sciences, MSc “Social – Preventive Medicine and Quality in Health Care”, Aristotle University of Thessaloniki, Thessaloniki, Greece; 3 4th Department of Pediatrics, School of Medicine, Aristotle University, Papageorgiou General Hospital, Thessaloniki, Greece; 4 Department of Hygiene, Social-Preventive Medicine and Medical Statistics, School of Medicine, Aristotle University of Thessaloniki, Thessaloniki, Greece

**Keywords:** Continuous glucose monitoring, Duty shifts, Healthy individuals, Glycemic control, Glucose metabolism, Paediatric residents

## Abstract

**Purpose:**

Glucose values in healthy humans are influenced by dietary habits, stress, hormonal signaling and lack of adequate sleep. Our aim was to quantify glucose fluctuations in healthy paediatric residents with the use of commercial continuous glucose monitoring systems(CGMS).

**Methods:**

Eligibility criteria were: i)participation in the full on-call duty schedule, ii)age: 25–40 years, iii)normal body mass index (BMI) iv)absence of chronic disease or treatment affecting glucose metabolism and v)signed informed consent.

**Results:**

Fifteen sensors were placed in 13 residents (8 females). They were monitored for 35 days of 24-hour duty shift in the Emergency Department (ED), 35 days after a 24-hour ED shift and 114 days away from 24-hour ED shift (regular 8-hour shift days). Time in normoglycemia (70–140 mg/dl) was lower and time in higher glucose ranges (141–180 mg/dl and 181–250 mg/dl) was higher during ED duty shifts. Most participants showed a significant increase in mean glucose during 24-hour ED duty shifts (97.67 ± 10.84 mg/dl) compared to days after ED duty (94.68 ± 10.16 mg/dl, *p* = 0.002) and regular working days (97.85 ± 10.08 mg/dl, *p* < 0.001). Glucose variability, assessed by coefficient of variation, was also higher during ED duty days (15.78) than on days after ED duty (13.85) and regular days (12.91). Night-time glucose (00.00–06.00) was similarly elevated during ED duty shifts (98.85 ± 11.85 mg/dl) compared to nights after ED duty (90.59 ± 10.99 mg/dl, *p* = 0.002) and nights on regular working days (90.72 ± 10.06 mg/dl, *p* = 0.003).

**Conclusions:**

24-hour ED duty shifts significantly affect glucose values of Paediatric residents. Further studies are warranted to confirm and further investigate these preliminary data.

## Introduction

Blood glucose levels show a normal variability during the day in healthy individuals, influenced by factors such as dietary habits, physical activity, stress, hormonal signaling and lack of adequate sleep. Several studies have demonstrated an impact on glucose patterns caused by even short-term changes in daily habits. Consumption of low-carbohydrates/high-fat meals for one day led to increased postprandial glucose on subsequent normal-carbohydrates meals [1], whereas short-term sleep loss resulted in statistically less physical activity in healthy men, with possible negative impact on metabolic health [2].

Rotational shift work has also been recognized as a disrupting factor for the circadian rhythm, which can predispose to obesity and impaired glucose tolerance [3]. In a study of the glucose metabolism of 12 nurses, higher values of postprandial glucose were observed during night shifts [4]. When metabolism of healthcare workers with and without night shift duties were compared, rotational night shifts were found to possibly have a negative impact on postprandial triglyceride responses and insulin sensitivity [5]. Similar results were found by Morris et al. when they studied glucose tolerance in long-term shift workers [6].

In recent years, the use of continuous glucose monitoring (CGM) systems has significantly improved glycemic control of people with type 1 diabetes mellitus, and has also helped to acquire valuable data on glucose metabolism in other diseases and in healthy populations [7]. Continuous glucose monitoring has showed that 96% of glucose values of nondiabetic people range between 70 and 140 mg/dl during a 24-hour period, while at night (00.00–06.00), they drop further and the time in this range reaches 99% [8]. Moreover, glycemic variability assessed using CGM in individuals without diabetes has been systematically reviewed and associated with cardiometabolic risk markers, highlighting the relevance of monitoring glucose fluctuations even in apparently healthy populations [9]. In parallel, advances in digital health and artificial intelligence are increasingly recognized as important tools for the early detection and prevention of dysglycemia, enabling the identification of subtle metabolic alterations before disease develops [10].

Paediatric residents in a tertiary hospital are highly influenced by stress factors during their 24-hour ED duty shift, staying for 24-hour stay in the hospital, being deprived of sleep, having irregular meals, as well as experiencing physical and mental exhaustion. This study aimed to capture the possible glucose fluctuations in pediatric residents, using CGM systems, and to compare measurements performed during 24-hour ED duty days with those performed on the days immediately after the 24-hour ED duty and on regular working days.

## Methods

### Participants

We conducted a prospective cohort study on glucose fluctuations of paediatric residents with normal glucose metabolism in a tertiary hospital. Inclusion criteria were: (i) participation in the full on-call duty schedule of a Paediatric Department, (ii) age: 25–40 years, (iii) normal body mass index (BMI), as calculated at study entry for all participants (iv) absence of chronic disease or treatment affecting glucose metabolism and (v) willingness to participate in the study after providing signed informed consent.

Normal glucose metabolism was assessed based on medical history, including the absence of known diabetes, prediabetes, or gestational diabetes and the absence of medications affecting glucose metabolism, and was further supported by CGM-derived metrics during the study, including normal estimated HbA1c and absence of pathological fasting or postprandial glucose values.

### Glucose fluctuations measurements

Two different CGM devices, Dexcom One and Freestyle Libre 1, were used for this study, according to the manufacturers’ instructions. They were placed on the upper arm of the participants and were allowed a warm-up period as recommended. Glucose data were available in real time either through a smartphone application or a dedicated reader device. The appropriate sensor placement interval was chosen for each participant, to cover the maximum number of 24-hour ED shifts (typically 2 or 3 ED shifts) during the given lifetime of the sensor (ten days for Dexcom one and fourteen days for Freestyle Libre 1). Glucose values were stratified in three groups: “during” 24-h ED duty shifts, 24-h “after” ED duty shifts and regular 8-hour shift days, “away” from ED duty shifts. Finally, we studied separately the “day-time” and “night-time” periods, we compared data from male and female participants and we analyzed data from each participant individually. Any readings during sensor startup or identified compression artifacts were excluded from the analysis. Missing or invalid data were minimal. It should be noted that off-duty days were not classified separately, as they were included either within the days “after” 24-hour ED duty or within the “regular/away” category.

### Primary and secondary outcomes

The primary outcome of the study was the distribution of glucose values across predefined glycaemic ranges, expressed as time below range (< 70 mg/dl), time in normoglycaemia (70–140 mg/dl), time in range 141–180 mg/dl and time above 180 mg/dl, comparing 24-hour ED duty days with days after and away from ED duty. Secondary outcomes included mean 24-hour and nocturnal glucose values, coefficients of variation, sex-specific comparisons, individual-level analyses, and day–night glucose differences among the three groups (“during”, “after” and “away” from ED duty days).

### Statistical analysis

The study data were initially collected and recorded in a worksheet package and then statistically analyzed using Microsoft® Excel for Mac, version 16.85 and IBM® SPSS Statistics version 29.0.1.0 for Mac. These software packages were used for both statistical analysis and graphical visualization of the results.

The normality of samples was tested with the Shapiro-Wilk test for samples less than 50 and with the Kolmogorov-Smirnov test for samples greater than 50. For parameters with normal distribution, the Student’s T-test was used to compare the means of two different groups and the Anova test was used to compare the means of more than two groups. In cases of non-normal distribution, Mann-Whitney and Kruskal-Wallis tests were used to compare means or more than two means. To compare rates the chi-square test was used. The limit of statistical significance was set to 0.05 (*p* < 0.05).

## Results

In total 13 paediatric residents were included in the study, 61.5% (*n* = 8) were women. The mean age of participants was 29.22 years, with a mean BMI of 22.51 kg/m² (23.55 kg/m² in men vs. 21.86 kg/m² in women). Six residents were in their third year of training and seven were in their fourth year. Fifteen sensors were placed, because there was a double placement in two female participants. Overall, there were 5 placements of Dexcom One (2 men and 3 women) and 10 placements of FreeStyle Libre 1 (3 men and 6 women). One woman used both CGM devices, whereas another one used the FreeStyle Libre 1 sensor twice. A total of 23,975 glucose measurements were recorded and classified in the following groups as described in Methods, (a) 35 ED duty days (5,034 readings), (b) 35 “after” ED duty days (4,636 readings) and (c) 114 regular working days i.e. “away” from ED duty days (14,305 readings). These measurements correspond to an average of 2.33 ± 0.82 duty days (range: 1–4 duty days) per sensor placement (each lasting 10 or 14 days).

### Primary outcome: distribution of glucose values across predefined glycaemic ranges

#### Analysis of data during the entire 24-hour period

**Fig. 1:**
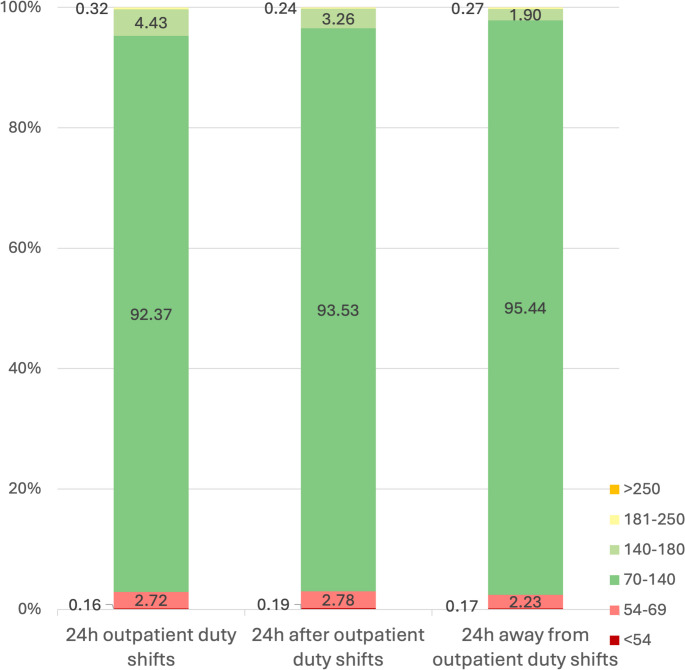
Significantly lower time in normoglycemia (TING, 70–140 mg/dl) and higher time in the interval 141–180 mg/dl on 24 h during ED duty shifts compared to 24 h “after” and 24 h “away” from ED duty shifts, in total (*p* < 0.001)

The distribution of all glucose values, classified into the predefined intervals and according to “ED duty”, “after ED duty” and “away from ED duty” days, is depicted in Fig. 1. A statistically significant difference in rates was recorded among the 3 categories with ED duty days having the lower rates of time in normoglycemia range (TING, 70–140 mg/dl) and the higher rates of time in the interval 141–180 mg/dl (*p* < 0.001) (Fig. 1).

#### Analysis of data during night hours (00.00–06.00)

**Fig. 2:**
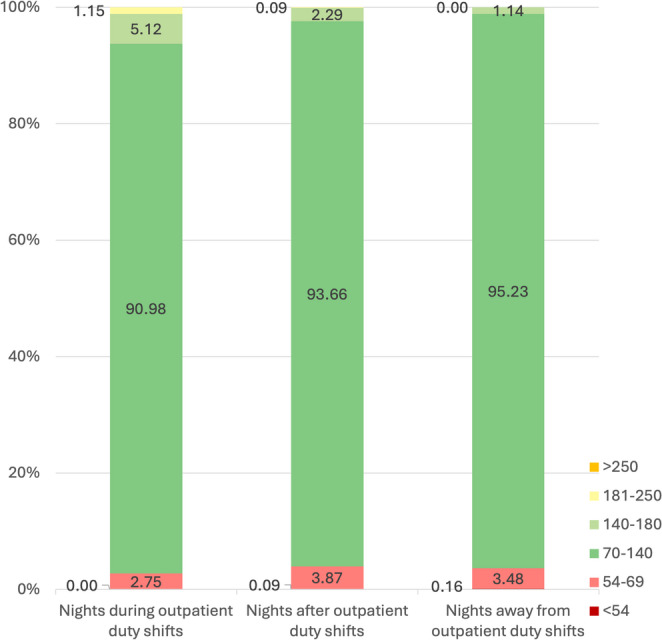
Significantly lower time in normoglycemia (TING, 70–140 mg/dl) and higher time in the interval 141–180 mg/dl on nights (00.00–06.00) during ED duty shifts compared to nights “after” and nights “away” ED duty shifts, in total (*p* < 0.001)

The distribution of the total glucose values classified in the predefined intervals and according to nights during ED duty shifts, nights “after” and nights “away” from ED duty shifts, is shown in Fig. 2. A statistically significant difference in rates was recorded among the 3 categories, with the nights during ED duty recording lower rates of time in normoglycemia (70–140 mg/dl) and higher rates in the interval 141–180 mg/dl, but also in the interval 181–250 mg/dl (*p* < 0.001) (Fig. 2).

### Secondary outcomes

#### Mean glucose values and glucose variability (Table 1)

**Table 1:** Mean glucose values and coefficient of variations on 24h and nights of ED duty shifts, nights “after” ED duty shifts and nights “away” from ED duty shifts

	24h ED duty	24h after	24h away
n (days)	35	35	114
Glucose (mg/dl)(mean value ± SD)	97.67 ± 10.84	94.68 ± 10.16^*1^	93.85 ± 10.08^*2^
CV(mean value ± SD)	13.85 ± 3.88	15.78 ± 5.22^*3^	12.91 ± 3.60^*4,*5^
	Night ED duty	Night after	Night away
n (nights)	35	35	114
Glucose (mg/dl)(mean value ± SD)	98.85 ± 11.85	90.59 ± 10.99^*6^	90.72 ± 10.06^*7^
CV(mean value ± SD)	12.92 ± 4.75	8.95 ± 3.22^*8^	9.43 ± 2.24^*9^

*1*p*=0.002 compared to 24h ED duty,, *2*p*<0.001 compared to 24h ED duty, *3*p*=0.035 compared to 24h ED duty, *4*p*=0.027 compared to 24h ED duty, *5*p*<0.001 compared to 24h after ED duty, *6*p*=0.002 compared to night on ED duty,, *7*p*=0.003 compared to night on ED duty, *8*p*=0.030 compared to night on ED duty,, *9*p*=0.016 compared to night on ED duty

For the succeeding statistical analysis, mean daily glucose and CV values were calculated and then for each resident mean values were calculated individually. Mean glucose values were higher on ED duty days (97.67 ± 10.84 mg/dl), compared to the days “after” ED duty days (94.68 ± 10.16 mg/dl, *p* = 0.002) and to days “away” from ED duty days (97.85 ± 10.08 mg/dl, *p* < 0.001). In addition, the coefficient of variation (CV) of glucose values was higher on days “after” ED duty compared to the CV of glucose measurements on ED duty days and to the CV on regular working days, “away” from ED duty (15.78 ± 5.22 vs. 13.85 ± 3.88, *p* = 0.035 and 12.91 ± 3.60, *p* < 0.001 respectively). Glucose values during the night hours (00.00–06.00) were analyzed separately. They were statistically higher on nights during ED duty (98.85 ± 11.85 mg/dl) compared to nights “after” (90.59 ± 10.99 mg/dl, *p* = 0.002) and nights “away” from ED duty (90.72 ± 10.06 mg/dl, *p* = 0.003). Additionally, CV was significantly higher on nights during ED duty (12.92 ± 4.75 mg/dl) compared to nights “after” (8.95 ± 3.22 mg/dl, *p* = 0.03) and nights “away” from ED duty (9.43 ± 2.24 mg/dl, *p* = 0.016).

#### Analysis of data per sex (Table 2)

**Table 2:** Classification of glucose values by sex, in nights of ED duty shifts, nights “after” ED duty shifts and nights “away” from ED duty shifts

	Males (*n* = 5)	Females (*n* = 8)	*p*
24 h of ED duty shifts			
n (days)	8	27	
Glucose (mg/dl) (mean value ± SD)	100.36 ± 6.80	96.00 ± 12.91	0.504
CV (mean value ± SD)	15.14 ± 3.89	13.04 ± 3.90	0.366
24 h after ED duty shifts			
n (days)	9	26	
Glucose (mg/dl) (mean value ± SD)	95.27 ± 6.90	94.31 ± 12.23	0.878
CV (mean value ± SD)	19.43 ± 5.55	13.50 ± 3.75	**0.040**
24 h away from ED duty shifts			
n (days)	44	70	
Glucose (mg/dl) (mean value ± SD)	96.32 ± 4.42	92.53 ± 12.49	0.509
CV (mean value ± SD)	14.69 ± 4.80	11.80 ± 2.30	0.167
Nights of ED duty shifts			
n (measurements)	8	27	
Glucose (mg/dl) (mean value ± SD)	100.97 ± 7.31	97.53 ± 14.32	0.632
CV (mean value ± SD)	16.02 ± 5.80	10.99 ± 2.88	0.059
Nights after ED duty shifts			
n (measurements)	9	26	
Glucose (mg/dl) (mean value ± SD)	90.78 ± 7.87	90.46 ± 13.10	0.962
CV (mean value ± SD)	8.98 ± 4.29	8.94 ± 2.69	0.980
Nights away from ED duty shifts			
n (measurements)	44	70	
Glucose (mg/dl) (mean value ± SD)	91.46 ± 6.39	90.26 ± 12.23	0.845
CV(mean value ± SD)	9.66 ± 1.32	9.28 ± 2.75	0.785

When glucose values were classified by sex, men showed higher mean glucose values on all instances, however without reaching statistical significances. CV of glucose values on days “after” ED duty were higher in man compared to woman (19.43 ± 5.55 vs. 13.50 ± 3.75, *p* = 0.040). When glucose values during night hours (00.00–06.00) were classified by sex, no statistically significant differences were observed.

#### Analysis of data per individual

**Fig. 3:**
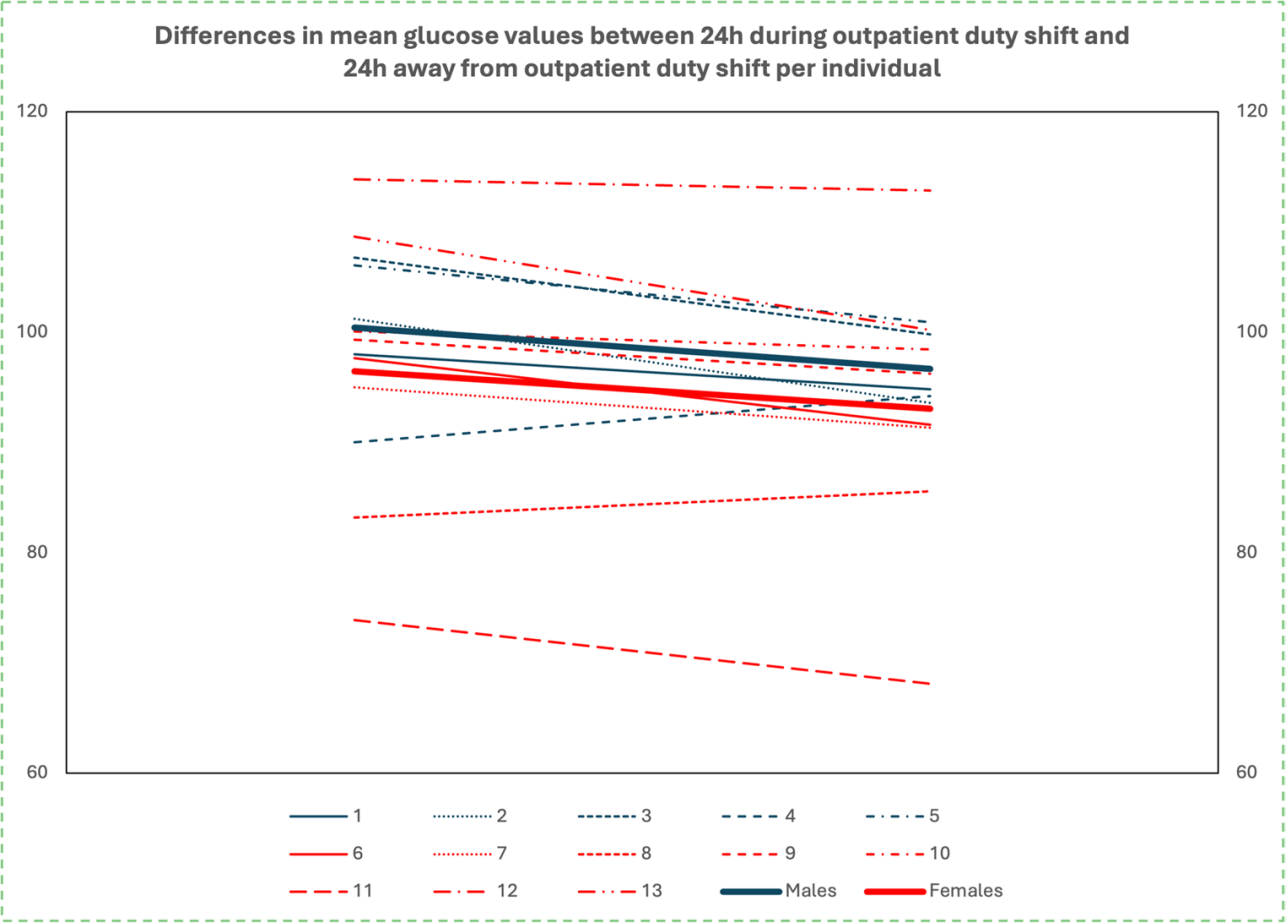
Mean glucose changes per resident individually and per sex from days during ED duty shift to days “away” from ED duty shift

When data were analyzed individually, most residents showed a statistically significant increase in average glucose values during days of ED duty shifts. There were only two participants, one male and one female, who, on the contrary, showed a statistically significant decrease in their average glucose compared to days “away” from ED duty, as well as one female participant, whose difference in average glucose values was not statistically significant (Fig. 3).

**Fig. 4:**
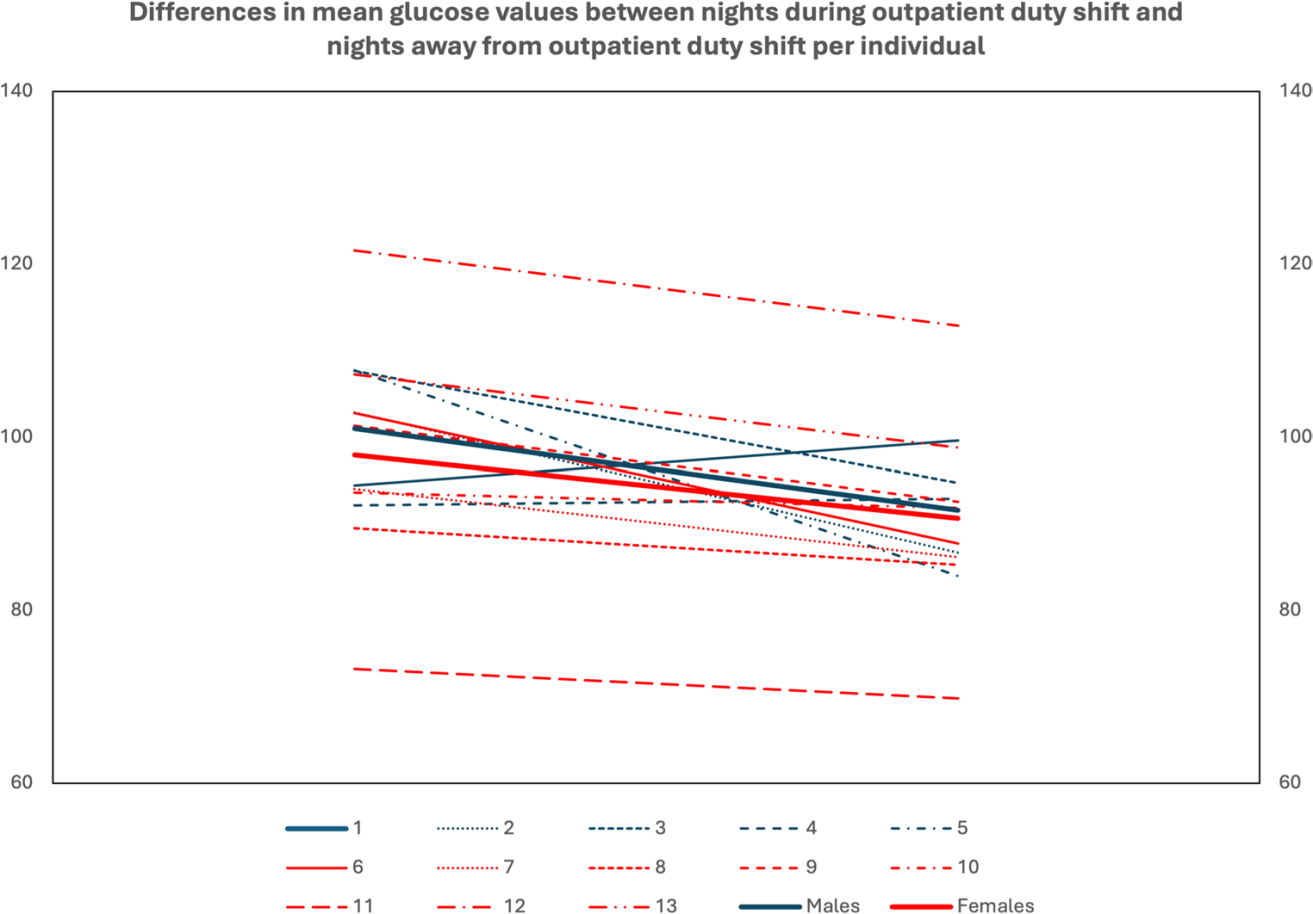
Mean glucose changes per resident individually and per sex from night during ED duty shift to night “away” from ED duty shift

Similarly, most residents showed a statistically significant increase in average glucose values of nights (00.00–06.00) during ED duty shifts compared to nights “away” from ED duty shifts. Only two male participants had higher average glucose at nights “away” from ED duty shifts and, in fact, in one of them this increase was statistically significant. Additionally, in a female participant, the increase of glucose values at nights during ED duty shifts did not reach statistical significance (Fig. 4).

Finally, the mean difference between 24 h glucose values and night glucose values [Δ (24 h glucose – night glucose)] was negative for ED duty shifts days and positive for regular working days, with a statistically significant difference between the two groups (-1.12 ± 3.90 vs. 3.43 ± 5.27, *p* = 0.013). The comparison of these differences in relation to gender showed a statistically significant difference only for female subjects (*p* = 0.017) (Table 3).

**Table 3:** Comparison of mean difference between day glucose values and night glucose values during ED duty shifts and regular working days in total and categorized by sex

	Δ (24h glucose – night glucose) during ED duty shifts	Δ (24h glucose – night glucose) during regular day	*p*
Males	-0.57 ± 2.36	5.08 ± 7.98	0.13
Females	-1.46 ± 4.75	2.40 ± 2.85	0.017
p	0.375	0.198	
Total	-1.12 ± 3.90	3.43 ± 5.27	0.013

## Discussion

This original study demonstrated higher glucose levels as well as higher variability of glucose values among pediatric residents during their 24-hour ED duty shifts compared to regular days. Impaired beta-cell function has also been described in nurses during rotational shift work [4, 11]. These findings are concerning, given that healthcare workers represent a population that is vulnerable to metabolic risks, such as insulin resistance, hypertension, obesity and dyslipidemia, and are twice as likely to present metabolic syndrome according to a recent metanalysis by Sooriyaarachchi et al. [12]. Another metanalysis of 12 observational studies by Gan et al., also highlighted the increased prevalence of diabetes mellitus among shift workers [13].

The factors contributing to these glucose fluctuations and their possible long-term consequences are various. A well-acknowledged one is the disruption of biological rhythms, also known as chronodisruption, which happens most profoundly with shift work, where the sleep-wake cycle is disturbed. The key role played by molecular clock mechanisms in glucose homeostasis is thoroughly described by Kalsbeek et al. [14], and is confirmed in our study, where pediatric residents lack adequate sleep, and present higher glucose levels during night. The combination of decreased duration of sleep and routine extension of work activity during the night may also lead to alternating periods of eating/fasting and energy storage/utilization [15], which are also considered as additional factors increasing metabolic risks. Eating habits among doctors during 24-hour duty shifts are also known to be less healthy than those in regular days (irregular meals, possible lack of healthy options at canteens during night shifts). A detailed record of meals consumed during days of CGM use in our study would have been of great importance, however it was not included in our study protocol. Finally, the physical and mental fatigue from prolonged working hours is also a stress factor that could explain higher glucose levels in our study. It should be noted, however, that while these fluctuations were statistically significant in this cohort of healthy, young residents, their clinical implications are likely limited, and our findings should be considered primarily hypothesis-generating rather than indicative of long-term metabolic risk.

When data in our study were analyzed categorized by sex, no statistically significant differences were observed with the exception of variation of glucose values being higher on males on days “after” ED duty compared to women. However, and in all instances, men appeared to have increased glucose values compared to women but without reaching statistical significance. In the metanalysis of Gan et al., male shift workers were more prone to diabetes mellitus than females [13]. Several studies have suggested that androgens improve insulin sensitivity, whereas low levels of testosterone promote insulin resistance [16–18]. Moreover, it is known that testosterone secretion is controlled by circadian rhythm systems [19]. In our small pilot study, our findings could suggest that disruption of the circadian rhythm during a 24-hour duty shift might lead to impaired androgen secretion and increased risk of hyperglycemia in men. However, given the limited sample size and the exploratory nature of our analyses, our data cannot support definitive conclusions regarding sex-specific mechanisms. Moreover, further laboratory tests would be needed to investigate all potential underlying mechanisms to explain these gender differences.

Continuous glucose monitoring systems have brought about a revolution in the management of patients with diabetes requiring insulin but they have also been used in pivotal studies in order to investigate the glucose profile in several diseases as well as in healthy populations. The comparison our results with data from Shah’s et al., multicenter study on CGM profiles of nondiabetic participants is interesting [8]. In Shah’s study, healthy participants had a median time in tight range (70–140 mg/dl) of 96%, which was close to our pediatric residents’ median time in tight range on days “away” from ED shifts (95.44%). However, on days during ED duty shifts and days “after” an ED duty shift, time in tight range dropped to 92.37% and 93.53% respectively. Moreover, residents’ time percentage above 140 mg/dl was double in comparison to Shah’s participants on days during ED duty shifts (4.43% vs. 2.1%) and similar on regular working days (1.90% vs. 2.1%). The coefficient of variation of doctors’ measurements was also similar to Shah’s group on regular days (17.49% vs. 17%) but increased during days of ED duty shift to 20.37%.

Glycemia profiles of non-diabetic people have also been investigated with continuous glucose monitoring in a more recent study with over 1,000 participants by Spartano et al. [20]. It is impressive that normoglycemic people demonstrated approximately 12.1% of measurements above 140 mg/dl and 1.2% above 180 mg/dl. However, the mean age of participants was 58.5 years, including obese people, whereas in Shah’s study the population was relatively younger, and obesity was an exclusion criterion. Our study population resembled more to Shah’s participants.

Our study has certain limitations. It is a single-center study with a small number of participants. We did not record meals during on-call days, individual eating habits, physical activity, or sleep patterns. Τwo different CGM systems were used, so measurement variability between devices cannot be entirely excluded. However, the within-subject design is expected to minimize the impact of potential device-related differences. Moreover, the fact that the CGM devices were not blinded may have introduced a behavioural component, as real-time access to glucose data is known to influence short-term lifestyle choices, including meal timing, food selection, and activity patterns. More broadly, this aligns with the well-described “Hawthorne effect,” in which awareness of being monitored alters behaviour [21]. However, it is the first study of glucose metabolism profile in healthy doctors working in Greek hospitals and it could be a stimulus for further investigation of the long-term metabolic consequences of working conditions on their health.

## Conclusions

This study provides novel insights into glucose fluctuations among pediatric residents during 24-hour ED duty shifts. Our findings indicate that these demanding work schedules are associated with transient increases in glucose levels and greater glycemic variability, likely influenced by factors such as sleep deprivation, irregular meals, and physical and mental fatigue. These findings need to be confirmed and supported by more prospective and larger studies. Additionally, future studies could also investigate the long-term consequences of these glucose fluctuations on doctors’ health and propose prevention strategies, including recommendations for healthier eating habits during duty shifts, as well as strict regulations on the extent of working shifts.

## Data Availability

Data is available upon reasonable request.
